# In silico modification of suberoylanilide hydroxamic acid (SAHA) as potential inhibitor for class II histone deacetylase (HDAC)

**DOI:** 10.1186/1471-2105-12-S13-S23

**Published:** 2011-11-30

**Authors:** Usman SF Tambunan, N Bramantya, Arli A Parikesit

**Affiliations:** 1 Department of Chemistry, Faculty of Mathematics and Science, University of Indonesia, Depok, Indonesia

## Abstract

**Background:**

The cervical cancer is the second most prevalent cancer for the woman in the world. It is caused by the oncogenic human papilloma virus (HPV). The inhibition activity of histone deacetylase (HDAC) is a potential strategy for cancer therapy. Suberoylanilide hydroxamic acid (SAHA) is widely known as a low toxicity HDAC inhibitor. This research presents *in silico* SAHA modification by utilizing triazole, in order to obtain a better inhibitor. We conducted docking of the SAHA inhibitor and 12 modified versions to six class II HDAC enzymes, and then proceeded with drug scanning of each one of them.

**Results:**

The docking results show that the 12 modified inhibitors have much better binding affinity and inhibition potential than SAHA. Based on drug scan analysis, six of the modified inhibitors have robust pharmacological attributes, as revealed by drug likeness, drug score, oral bioavailability, and toxicity levels.

**Conclusions:**

The binding affinity, free energy and drug scan screening of the best inhibitors have shown that 1c and 2c modified inhibitors are the best ones to inhibit class II HDAC.

## Background

Cervical cancer is one of the most prevalent cancers for women, and it is the most prevalent one in developing countries. It is estimated that in the year 2000, there were 470,600 new cervical cancer cases, with 233,400 deaths. Moreover, 80 % of these cases happened in developing countries [1]. In Indonesia, it is estimated, that there are 100 new cervical cancer cases per 100,000 people. It is known that 70% of them are in the late stages [2].

Cervical cancer occurs at the area known as the cervix. The cause of this cancer is the human papilloma virus (HPV), a member of the *Papillomaviridae* family. More than 120 types of HPV have been identified, and out of that number, 15 of them are classified as high risk HPV types (16, 18, 31, 33, 35, 39, 45, 51, 52, 56, 58, 59, 68, 73, and 82) with 12 of them being low risk HPV types (6, 11, 40, 42, 43, 44, 54, 61, 70, 72, 81, and CP6108). Types 16 and 18 are the main cause of the 70% of cervical cancer case, while 41-54% caused by Type 16 HPV alone [3]. The most effective and safe method for tackling HPV infection is still not available, with treatments options being surgery and/or with physico or chemotherapy [4].

The inhibition of histone deacetylase (HDAC) activity, which is manifested by the destruction of HDAC complex, has been widely known as a potent measure to combat cervical cancer. HDAC (EC 3.5.1) is the enzyme, which catalyzes the histone deacetylation within eukaryotes. Deacetylation is a release of the acetyl group from the histone tail, and it causes the histone to be twisted around the DNA, disrupting gene transcription, by blocking the pathway of transcription factor binding [5]. The inhibition of HDAC by its specific inhibitor shows a couple of changes at the molecular and cellular level [6]. The HDAC activity inhibition by specific inhibitors could induce the death of the cancer cell [5].

Vorinostat or suberoylanilide hydroxamic acid (SAHA) is the most widely used inhibitor of class II HDAC activity. This inhibitor has carbonyl and hydroxylamine groups, which will bind to the zinc ion, Zn^2+^ , with the aliphatic chain as linker, and the hydrophobic group in the other tail. Our research group has successfully determined the efficacy of SAHA as a potential class II HDAC inhibitor [4].

We are looking to compare the efficacy of SAHA with other types of inhibitors, by searching for the new ones or modifications of the existing ones. Triazole is known as a non-classical amide bioisostere compound [7]. Triazole could replace the amide bond in the SAHA side group without losing its activity significantly [8].

We are interested to modify the SAHA compound, by creating new ones. The processes we have followed are replacing one of the amides group within the SAHA hydrophobic group with triazole, and adding triazole as hydrophobic group toward SAHA. Then, we conduct molecular docking with Class II HDAC, and testing its toxicity, and finally compare the result with standard SAHA inhibitor. This structure-activity relationship (SAR) study is very important in uncovering novel inhibitors of HDAC.

## Material and methods

### Collecting the *Homo sapiens* Class II HDAC sequences and its 3D structure

Collecting of *Homo sapiens* Class II HDAC sequences was done by downloading them from the protein database at NCBI site (http://www.ncbi.nlm.nih.gov). The *Homo sapiens* Class II HDAC 3D crystal structure was downloaded from the PDB structural database site (http://www.rcsb.org/pdb). The sequences were analyzed to determine whether there are any newly curated sequences or not.

### Sequence conservation at the *Homo sapiens* class II HDAC catalytic site

ClustalW multiple sequence alignment of the collected *Homo sapiens* Class II HDAC sequences was carried out. The alignment results were analyzed with BioEdit, in order to obtain the existing catalytic site. The conserved region information was the result of the *Homo sapiens* Class II HDAC sequence alignment with its 3D structure sequences. The sequence for modelling will be the one that is closely resembles the crystal structure of *Homo sapiens* Class II HDAC. This research follows on from the previous research, which used Class II HDAC *Homo sapiens *[4]. If the result of the sequence alignment is the same with the previous ones, the Class II HDAC *Homo sapiens* structure will be generated from it, without the necessity of conducting another homology modelling process.

### Design of SAHA and modified SAHA inhibitors

This is the beginning of SAR study. The structure of SAHA and modified SAHA were designed by using ChemSketch 12.0 software. Various SAHA modifications were utilized. The output of ChemSketch 12.0 is in the *mol* format. It functions as an input for docking simulations with class II HDAC of *Homo sapiens*, and to test the pharmacology and toxicity attributes. The ligands were saved in MDL Molfile format. Then, they were converted to pdb format by using OpenBabel 2.2.3 or Vegazz software.

### Preparation of class II HDAC *Homo sapiens* docking

Class II HDAC *Homo sapiens* structure files were prepared in pdb format. Then, its variations: HDAC 4, 5, 6, 7, 9, and 10 were loaded with AutoDock Tools, and polar hydrogen was added to each of those HDAC. The addition is useful for giving the partial charges/gasteiger charges to those enzymes. Then, they were saved in pdbqt format. Then, the Zn charge of class II HDAC *Homo sapiens* was converted from 0 to +2 by using python script. Then, the molecule was adjusted as macromolecule for the docking process.

### Preparation of class II HDAC *Homo sapiens* inhibitor file

The inhibitors or ligands in pdb format were loaded with AutoDock Tools software. Then, the torsion of the ligand was adjusted based on the total number of rotatable bonds. Ligands were saved in pdbqt format.

### Grid box preparation

The preparation steps were started by using pdb file of *Homo sapiens* Class II HDAC as the receptor, and SAHA with its various ligand modifications. Grid Box is the coordinate area determination for the docking process. It is configured in AutoDock Tools. The grid box size for the docking of HDAC 4, HDAC 5, HDAC 6, HDAC 7, HDAC 9, and HDAC 10 with the ligands are consecutively 19.228, -6.296, 0.177; 19.696, -6.093, 0.513; 15.326, -7.608, 8.495; 18.484, -6.198, -0.744; 18.078, -7.314, -2.129 and 17.713, -6.707, 1.288, with the spacing between grid points of 0.375 Å. We obtained those numbers from our previous research [4]. The best docking model result was then picked. The grid box was saved in a grid parameter file (gpf) format.

### Docking simulation

This process was done using AutoGrid 4.2 and AutoDock 4.2. The following data are necessary for conducting the docking: enzyme file in pdbqt format, ligand in pdbqt format, gpf files, dpf files. The utilized algorithm is Lamarckian Genetic Algorithm (LGA) with the population size of 150, energy evaluation of 2.5 x 10^6^ and search runs of 100 times within the RMSD of 1.5.

### Analysis and visualization of docking simulation results

The docking result of AutoDock 4.2 is in the docking log file (dlg) format. Then, by using python script, the docking results were converted to pdb format. Out of 100-model result, one best model was picked up, based on the free energy bonding data, in order to analyze its interaction.

### Drug scan

This was conducted in order to determine, whether the inhibitor has fulfilled the conditions as the drug candidate based on Lipinski’s Rule of Five. It is done using Lipinski Filters, Molinspiration, Osiris Property Explorer, Toxtree v2.1.0 and Lazar software.

Molinspiration and Lipinski Filters were utilized for analyzing the molecular attributes, such as Log P, the amount of hydrogen bond donors, the amount of hydrogen bond acceptor, and the molecular mass of the drugs. Moreover, the Osiris Property Explorer, Toxtree v2.1.0, and Lazar calculated various attributes of the drugs, such as toxicity, drug likeness, and drug score.

In order to use Lipinski Filters, the ligand in pdb format must be uploaded to the analysis software website. The same applies to Molinspiration, Lazar, and Toxtree v2.1.0, because the ligand in smiles format must be uploaded to their websites. However, to use Osiris Property Explorer, the ligand could be drawn offline.

## Results and discussion

### Determination of class II HDAC *Homo sapiens*

The results of the class II HDAC *Homo sapiens* search in the NCBI sites are 65 sequences shown in Table 1.

**Table 1:** Class II HDAC Homo sapiens sequence search result.

Enzyme	Sequence amount	Sequence code
HDAC 4	5	[Genbank: NP_006028.2, Genbank:AAD29046.1, Genbank:EAW71166.1, Genbank:EAW71165.1, Genbank:P56524.3]

HDAC 5	7	[Genbank: AAD29047.1, Genbank: Q9UQL6.2, Genbank: AAH51824.1, Genbank: NP_001015053.1, Genbank: NP_005465.2; EAW51634.1, Genbank: EAW51633.1]

HDAC 6	9	{Genbank:NP_006035.2, Genbank: AAD29048.1, Genbank: AAP35295.1, Genbank: Q9UBN7.2, Genbank: EAW50748.1, Genbank: EAW50747.1, Genbank: EAW50746.1, Genbank: EAW50745.1, Genbank: EAW50744.1}

HDAC 7	4	[Genbank:AAF63491.1, Genbank: Q8WUI4.2, Genbank: NP_056216.2, Genbank: NP_001091886.1]

HDAC 9	20	[Genbank:AAI52406.1, Genbank: AAI50329.1, Genbank: AAI11736.1, Genbank: NP_848512.1, Genbank: NP_848510.1, Genbank: NP_478056.1, Genbank: NP_055522.1, Genbank: AAO27363.1, Genbank: AAK66821.1, Genbank: Q9UKV0.2, Genbank: EAW93706.1, Genbank: EAW93705.1, Genbank: EAW93704.1, Genbank: EAW93710.1, Genbank: EAW93709.1, Genbank: EAW93708.1, Genbank: EAW93707.1, Genbank: EAW93703.1, Genbank: EAW93702.1, Genbank: EAW93701.1]

HDAC 10	20	[Genbank:AAL30513.1, Genbank: AAI25084.1, Genbank: AAS48345.1, Genbank: Q969S8.10, Genbank: NP_001152758.1, Genbank: NP_114408.3, Genbank: AAK92206.1, Genbank: AAK92205.1, Genbank: AAK84023.1, Genbank: EAW73519.1, Genbank: EAW73518.1, Genbank: EAW73515.1, Genbank: EAW73514.1, Genbank: EAW73513.1, Genbank: EAW73512.1, Genbank: EAW73511.1, Genbank: EAW73520.1, Genbank: EAW73517.1, Genbank: EAW73516.1, Genbank: BAD92656.1]

**Figure 1:**
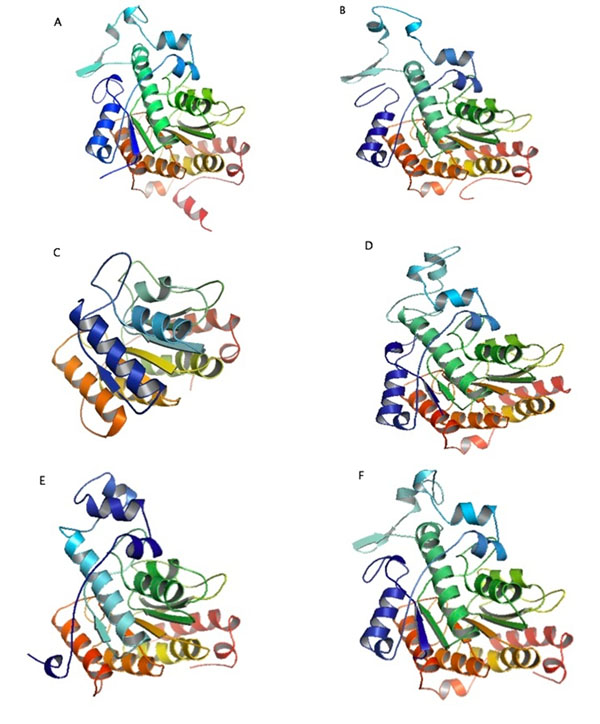
**Catalytic regions of the crystal structure of HDAC enzymes.** A. HDAC 4 *Homo sapiens.* B. HDAC 5 *Homo sapiens.* C. HDAC 6 *Homo sapiens.* D. HDAC 7 *Homo sapiens.* E. HDAC 9 *Homo sapiens.* F. HDAC 10 *Homo sapiens.* The catalytic regions of the 3D structure of class II HDAC *Homo sapiens* shown are based on their conserved regions.

Those sequences were downloaded in FASTA format, in order to conduct multiple sequences alignment for each class II HDAC *Homo sapiens*. The ones with the highest score are chosen as the modelling sequences. The alignment results were compared with the class II HDAC *Homo sapience* sequence from previous research [4]. The obtained sequences for HDAC 4, HDAC 5, HDAC 6, HDAC 7, HDAC 9, and HDAC 10 are [Genbank:NP_006028.2, Genbank:NP_005465.2, Genbank:NP_006035.2, Genbank:NP_056216.2, Genbank:NP_848510.1, and Genbank:NP_114408.3] consecutively. These sequences are the same as the sequences from our previous research [4]. Henceforth, this research utilized the 3D structure of class II HDAC *Homo sapiens* without the need to redo the homology modelling. Class II HDAC *Homo sapiens* has a long chain length (over than 1000 amino acid). In that case, the modelling was conducted by extracting the conserved region. The 3D structure of class II HDAC *Homo sapiens* are the catalytic area based on its conserved region [4], shown in Figure 1.

### The visualization of the class II HDAC *Homo sapiens* active site

**Figure 2:**
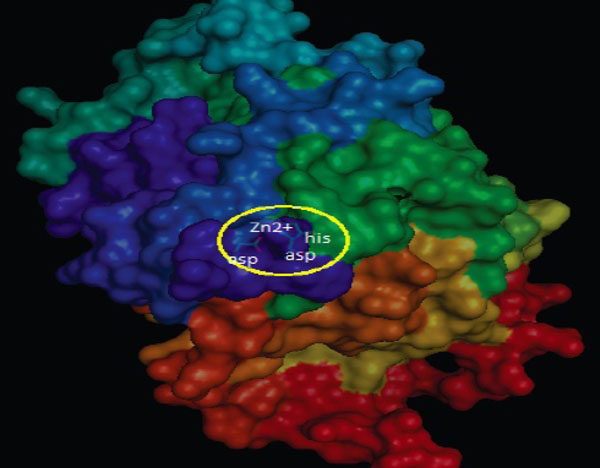
**The visualization of class II HDAC *Homo sapiens* active site.** PyMol visualization shows, that the utilized enzyme has cofactor Zn^2+^ as catalytic site, which is bound to three amino acid (two aspartic acid and one histidine) residues.

The 3D structure of class II HDAC *Homo sapiens* modelling results are saved in pdb format. PyMol visualization (Figure 2) shows, that the enzyme has cofactor Zn^2+^ as catalytic site, which is bound to three amino acid residues. These are two aspartic acids and one histidine residue.

The position of the catalytic sites of each class II HDAC is different. Table 2 shows the positions of the catalytic sites of HDAC4, HDAC 5, HDAC 6, HDAC7, HDAC9, and HDAC 10.

**Table 2:** Location of class II HDAC *Homo sapiens* catalytic sites.

Enzyme	Location of catalytic site
HDAC 4	Ion Zn^2+^ dengan residu Asp193, His195, and Asp287
HDAC 5	Ion Zn^2+^ dengan residu Asp178, His180, and Asp272
HDAC 6	Ion Zn^2+^ dengan residu Asp71, His73, and Asp164
HDAC 7	Ion Zn^2+^ dengan residu Asp191, His193, and Asp285
HDAC 9	Ion Zn^2+^ dengan residu Asp188, His190, and Asp282
HDAC 10	Ion Zn^2+^ dengan residu Asp133, His135, and Asp226

### The Design of class II HDAC *Homo sapiens* inhibitors

**Figure 3:**
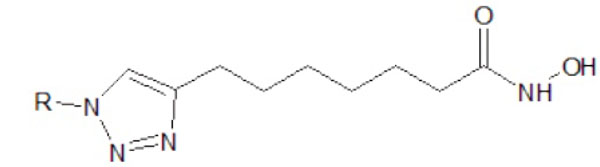
**The first modification of SAHA ligand.** This utilises triazole as substituent of amide group at SAHA.

**Figure 4:**
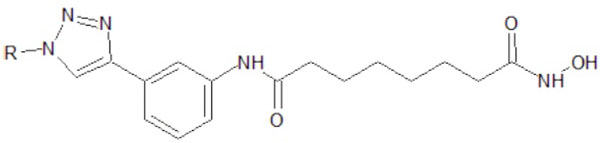
**The second modification of SAHA ligand.** This utilises triazole as additional group of SAHA, without substituting the amide group.

**Figure 5:**
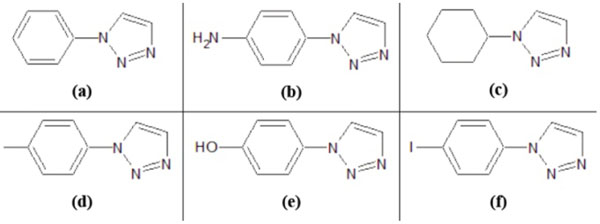
**The modified alkyl group.** Both modifications in figures 3 and 4 are made in their alkyl (R) groups.

12 modified ligands and one standard ligand were drawn by using ACDLabs ChemSketch 12.0. Those 12 modified ligands could be classified as two groups. The first one utilises triazole as substituent of the amide group at SAHA (Figure 3). The second one utilises triazole as additional group of SAHA, without substituting the amide group (Figure 4). Both modifications are made in their alkyl groups (R groups) (Figure 5).

The triazole bioisostere attributes on SAHA amide group could eventually modify SAHA’s properties. The hydrophobic tendency of triazole compared with the amide group on SAHA was expected to increase the binding affinity of modified ligands toward class II HDAC *Homo sapiens*. Thus, the binding of enzyme-ligand complex would be much stronger [8]. Triazole could be treated as an additional functional group on SAHA, which could increase the hydrophobic attributes of SAHA cap group [9].

The six alkyl groups for modified ligand variations are phenyl (C_6_H_5_), phenylamine (C_6_H_4_NH_2_), cyclohexyl (C_6_H_11_), fluorophenyl (C_6_H_4_F), hydroxyphenyl (C_6_H_4_OH), and iodophenyl (C_6_H_4_I). The selections of those alkyl groups are based on hydrophobic attributes of those groups. Thus, this study would observe the influence of the cap group hydrophobicity of each modified ligand, in comparison with the SAHA standard ligand.

The design of the standard ligand and its modifications are conducted by clean structure procedure and 3D optimization with ChemSketsch 12.0. The objective of this treatment is to smooth the designed ligand structure, while the 3D optimization was conducted to visualize the ligand when the docking process is running.

### Preparation of the enzyme and ligand files for docking

The prepared files for the docking process are enzyme and ligand in pdbqt format, grid box parameter in gpf format, and docking parameter in dpf format. Pdbqt Format of enzyme and ligand show that both of them have undergone partial charge changes and atom type rearrangement. Zn with zero charge would not be recognized as a cofactor by the enzyme. Thus, Zn metal on class II HDAC *Homo sapiens* must undergo charge change to Zn^2+^ ion. This would be necessary for the ligand to interact with Zn^2+^ cofactor during the docking process.

When the ligands have been loaded with AutoDockTools, the amount of rotatable bonds were verified. The rotatable bonds are necessary to arrange the ligand torsional degree for docking process. If the amount of rotatable bonds and ligand torsional degree are increased, then the ligands would be more flexible, causing more variation in the docking process.

The grid box parameters are necessary to describe the docking process area. The docking parameters are needed to give necessary conditions in the docking process, and include the flexibility of enzyme, the selection of ligand, and the selection of the utilized algorithm.

### Docking

This process is of two types: blind and oriented docking. Blind docking is a docking process without knowing the position of the enzyme’s active site, and then during the grid box determination, the utilized grid center is not specific to the certain area, but applied to the whole enzyme area. Oriented docking was done by determine the position of the enzyme active site, and then the grid box could be specifically resolved. This research used oriented docking, because the active site region has been determined.

This docking process is semi-flexible, which means that the ligand was made flexible, while the enzyme was rigid. The docking was started by utilize autogrid with Autogrid 4.2 software. The objective is to map the area for docking process. The autogrid process was done using the determined grid box. The dimension of the utilized grid box must be big enough, in order that the ligand can be freely rotated [10]. The autogrid result in grid log file (glg) format would be applied as parameters for docking process.

This docking process used the Lamarckian Genetic Algorithm (LGA). This algorithm is recommended, because it is a hybridization of Local Search and Genetic Algorithm. The energy evaluation values and utilized search runs would have impacts on docking duration and docking energy values. The amounts of search runs are the total iteration or docking replication [10]. This study has conducted 100 iterations, and it would result in 100 inhibitor models on each docking.

### Interaction of the inhibitor with class II HDAC *Homo sapiens*

The docking results with AutoDock 4.2 were saved in dlg format. However, visualizing them in 3D graphic requires conversion to pdb format. The docking results in pdb can be visualized by PyMol software. One out of 100 models was taken as the best ligand mode, based on the best binding energy interaction between standard polar group ligand, and Zn^2+^ cofactor modification as enzyme catalytic site. The chosen interaction is the ligand model which has Zn^2+^ cofactor binding O atom on the C=O and –OH groups.

The docking result in this study shows, that the standard SAHA ligand and both modified ligands have equal amount of interaction toward Zn^2+^ cofactor. It is electrostatic attraction of both O atom on C=O and –OH functional groups toward Zn^2+^ cofactor. Moreover, the SAHA standard ligand and both modified ligands have hydrogen bonds with amino acid residues nearby the Zn^2+^ ion. This case made the binding interaction not applicable for determining which ligand has the best affinity toward Zn^2+^ . The interaction of SAHA standard ligand and both modified ligands toward class II HDAC *Homo sapiens* are presented in Tables 3 and 4.

**Table 3:** Interaction of SAHA standard ligand and first modified ligand with class II HDAC Homo sapiens.

Ligand	Docking interaction					
	
	HDAC 4	HDAC 5	HDAC 6	HDAC 7	HDAC 9	HDAC 10
SAHA	**Zn**^**2+**^**, Asp287,** His155, His 195	**Zn**^**2+**^**, Asp272,** His 140	**Zn**^**2+**^, His32, His33, Tyr204	**Zn**^**2+**^, **Asp191**, His153, His154	**Zn**^**2+**^, **Asp188**, His22, His150, His151	**Zn**^**2+**^, **Asp226**, His95, His96, Phe163, Tyr266

1a	**Zn**^**2+**^, **Asp287**, His155, Pro295	**Zn**^**2+**^, **Asp178**, His140, **His180**	**Zn**^**2+**^, His32, His33, Tyr204	**Zn**^**2+**^, **Asp191**, Glu115, His153	**Zn**^**2+**^, **Asp282**, His150, His151	**Zn**^**2+**^, **Asp133**, His95, His96, Tyr266

1b	**Zn**^**2+**^, **Asp193**, His155, Pro295	**Zn**^**2+**^, **Asp178**, His140, His141, Pro280	**Zn**^**2+**^, **Asp164**, His32, His33, Tyr204	**Zn**^**2+**^, Ala160, **Asp191**, Glu115, His153, His154, Thr109	**Zn**^**2+**^, Asp105, Asp107, **Asp188**, His150, His151	**Zn**^**2+**^, Asn103, **Asp133**, His95, His96

1c	**Zn**^**2+**^, **Asp193**, His155, Leu296	**Zn**^**2+**^, **Asp178**, His140	**Zn**^**2+**^, His32, His33, Thr100, Tyr204	**Zn**^**2+**^, **Asp191**, Glu115, His153, His154, Thr109	**Zn**^**2+**^, **Asp188**, **Asp282**, His150, His151	**Zn**^**2+**^, **Asp133**, His95, His96, Tyr226

1d	**Zn**^**2+**^, **Asp287**, His155	**Zn**^**2+**^, **Asp178**, His140, Glu102	**Zn**^**2+**^, His32, Thr100, Tyr204	**Zn**^**2+**^, **Asp285**, Glu115, His154, Thr109	**Zn**^**2+**^, **Asp188**, His150, His151, Pro290	**Zn**^**2+**^, **Asp133**, His95, His96

1e	**Zn**^**2+**^, **Asp193**, His155, Pro295	**Zn**^**2+**^, Glu102, Gly149, His140	**Zn**^**2+**^, **Asp164**, His32, His33, Pro103, Tyr204	**Zn**^**2+**^, Ala160, **Asp191,** Glu115, His153, Thr109, Thr111	**Zn**^**2+**^, Asp105, His150, His151	**Zn**^**2+**^, His95, His96, **Asp133**

1f	**Zn**^**2+**^, **Asp287**, Gly328, His155, Pro295	**Zn**^**2+**^, **Asp178**, Gu102, His140	**Zn**^**2+**^, **Asp164**, His32, His33, Thr100, Tyr204	**Zn**^**2+**^, Asp110, **Asp191**, His153, His154, Thr109, Thr111	**Zn**^**2+**^, **Asp188**, His150	**Zn**^**2+**^, **Asp133**, His95, His96

* Bold is the catalytic sites.

**Table 4:** Interaction of SAHA standard ligand and second modified ligand with class II HDAC Homo sapiens.

Ligand	Docking interaction					
	
	HDAC 4	HDAC 5	HDAC 6	HDAC 7	HDAC 9	HDAC 10
SAHA	**Zn**^**2+**^, **Asp287**, His155, His 195	**Zn**^**2+**^, **Asp272**, His 140	**Zn**^**2+**^, His32, His33, Tyr204	**Zn**^**2+**^, **Asp191**, His153, His154	**Zn**^**2+**^, **Asp188**, His22, His150, His151	**Zn**^**2+**^, **Asp226**, His95, His96, Phe163, Tyr266

2b	**Zn**^**2+**^, **Asp193**, Glu117, His155, Pro162, Tyr167	**Zn**^**2+**^, His140	**Zn**^**2+**^, Ala39, **Asp71**, His32, His33, Gly202	**Zn**^**2+**^, Ala160, Glu115, His153, Phe163, Thr109, Thr111	**Zn**^**2+**^, **Asp188**, His150, His151, Pro220	**Zn**^**2+**^, Asn103, **Asp133**, His95, Phe163

2c	**Zn**^**2+**^, **Asp193**, His155	**Zn**^**2+**^, **Asp178**, His140	**Zn**^**2+**^, His32, His33, Tyr204	**Zn**^**2+**^, **Asp191**, Glu115, His153, Thr109, Thr111	**Zn**^**2+**^, **Asp188**, Gly322, His150, His151	**Zn**^**2+**^, **Asp133**, His95, His96, Phe163

2d	**Zn**^**2+**^, **Asp193**, His155, Tyr167	**Zn**^**2+**^ ,**Asp178**, His140, His141, Glu102	**Zn**^**2+**^, His32, His33, Tyr204	**Zn**^**2+**^, His153, His154, Phe163	**Zn**^**2+**^, Asn217, **Asp188**, **His190**	**Zn**^**2+**^, Asn103, **Asp133**, His95, His96, Tyr266

2e	**Zn**^**2+**^, **Asp193**, His155	**Zn**^**2+**^, **Asp178**, His140, Tyr60	**Zn**^**2+**^, Gly99, His32, His33, Tyr204	**Zn**^**2+**^, **Asp285**, Glu115, Gly326	**Zn**^**2+**^, **Asp188**, His150, His151, **His190**, Ser19, Thr20	**Zn**^**2+**^, **Asp133**, Gln98, His96, Phe163

2f	**Zn**^**2+**^, **Asp193**, His155, **His195**	**Zn**^**2+**^, **Asp178**, His140, Phe209	**Zn**^**2+**^, His32, Thr100	**Zn**^**2+**^, Asp110, **Asp285**, His153, His154	**Zn**^**2+**^, **Asp188**, Asp217, Gly322, His150, His151	**Zn**^**2+**^, Asn103, **Asp133**, His95, His96,

* Bold is the catalytic sites..

### Binding free energy (ΔG_binding_) and inhibition constant (*K_i_*)

The results of the docking are the **ΔG_binding_** and *K_i_* values. The selection of AutoDock 4.2 best model ligand calculation result was based on the lowest binding free energy, and ligand interaction toward Zn^2+^ ion at the enzyme. The selection is not based on the cluster result. The values of the binding free energy and inhibition constant are available from Tables 5 and 6. The docking result shows, that all 12 modified ligands have lower binding free energy and inhibition constant values, compared with the SAHA standard ligand, for every enzyme in class II HDAC *Homo sapiens.* Ligand 2c has the smallest binding free energy and inhibition constant in HDAC 4 and HDAC 6. Ligand 2f has the smallest values for HDAC 5. Ligands 2d and 2f have the smallest values for HDAC 7. Last but not least, ligand 1c has the smallest values for HDAC 9 and HDAC 10.

**Table 5:** The binding free energy docking simulation result of SAHA standard ligand and its modification toward class II HDAC *Homo sapiens.*

Ligand	Binding energy, ΔG (kcal/mol)					
	
	HDAC 4	HDAC 5	HDAC 6	HDAC 7	HDAC 9	HDAC 10
SAHA	-7.21	-6.96	-7.19	-7.09	-6.49	-6.95
1a	-8.60	-7.90	-7.66	-7.81	-7.84	-9.10
1b	-8.08	-7.19	-7.01	-7.88	-7.29	-8.05
1c	-7.80	-7.68	-7.54	-8.47	**-8.23**	**-9.43**
1d	-7.89	-7.55	-7.41	-7.79	-7.51	-8.49
1e	-7.47	-7.50	-7.57	-7.63	-7.86	-8.25
1f	-9.06	-8.93	-8.10	-9.14	-7.46	-8.43
2a	-8.80	-9.60	-8.39	-9.07	-8.05	-8.31
2b	-9.38	-9.65	-7.58	-8.74	-7.20	-8.04
2c	**-9.44**	-8.87	**-9.75**	-9.24	-8.22	-7.94
2d	-8.72	-9.22	-8.22	**-9.27**	-7.88	-8.43
2e	-8.69	-9.82	-8.17	-8.67	-6.34	-8.13
2f	-9.04	**-10.63**	-8.50	**-9.27**	-7.86	-8.20

* Numbers in bold are the lowest amount of binding energy value.

**Table 6:** Inhibition constant result of standard ligand docking simulation and modification towards HDAC Class II.

Ligand	Inhibition constant, Ki (nM)					
	
	HDAC 4	HDAC 5	HDAC 6	HDAC 7	HDAC 9	HDAC 10
SAHA	5160	7940	5350	6400	17600	8100
1a	500	1620	2430	1880	1800	210
1b	1200	5350	7310	1690	4560	1260
1c	1900	2360	7540	610	**920**	**120**
1d	1660	2920	63110	1930	3140	590
1e	3360	3150	2820	2560	1740	890
1f	230	290	1150	200	3390	660
2a	360	90	710	220	1250	810
2b	130	80	2760	390	5300	1270
2c	**120**	310	**70**	170	940	1500
2d	400	180	940	**160**	1680	670
2e	430	630	1030	440	22380	1100
2f	240	**50**	590	**160**	1730	970

* Numbers in bold are the lowest amount of inhibition constant value.

The AutoDock values of **ΔG_binding_** in Table 5 show that every ligand has negative **ΔG.** It shows that the SAHA standard and modified ligand conformation complex with the tested HDAC, are much more stable than the individual conformations. It happens because binding releases energy, which is useful for decreasing the activation energy of catalytic reaction [4]. The negative binding free energy shows that the reaction is spontaneous. Tables 5 and 6 showed that the binding free energy values of each ligand are related to its inhibition constant values. The best ligand for each class II HDAC *Homo sapiens* has the smallest **ΔG_binding_** and *K_i_.*

### Pharmacology inhibition prediction

Molinspiration, Lipinski Filters, and Osiris Property Explorer were utilized to screen the drug candidate based on Lipinski’s Rule of Five and Oral Bioavailability. The prediction results of the pharmacological attributes are in Table 7.

**Table 7:** The molecular descriptor value of the SAHA standard ligand and modified ligand.

Ligand	Molecular weight	LogP	n ON^1^	n OHNH^2^	TPSA	n rotb	***Drug likeness***	***Drug score***
SAHA	264.325	2.467	5	3	78.42	8	-8.87	0.35
1a	288.351	2.391	6	3	80.04	8	-8.5	0.36
1b	303.366	1.467	7	4	106.07	8	-10.6	0.1
1c	294.399	3.022	6	3	80.04	8	-8.17	0.35
1d	306.341	2.555	6	4	80.04	8	-9.56	0.35
1e	304.35	1.912	7	4	100.27	8	-8.3	0.36
1f	414.247	3.474	6	3	80.04	8	-8.02	0.29
2a	407.474	3.306	8	4	109.14	10	-7.4	0.26
2b	422.489	2.382	9	5	135.17	10	-9.54	0.07
2c	413.522	3.937	8	4	109.14	10	-7.12	0.25
2d	425.464	3.469	8	5	109.14	10	-8.5	0.24
2e	423.473	2.826	9	5	129.37	10	-7.23	0.27
2f	533.37	4.389	8	4	109.14	10	-6.98	0.17

^1^ = number of hydrogen bonds

^2^= number of hydrogen bond acceptors

The parameters of Lipinski’s Rule of Five are as follows: the molecular weight must be less than 500 Da, LogP less than 5, the amount of Hydrogen donor (n OHNH) must be less than 5, the amount of acceptor hydrogen (n ON) must be less than 10, and the refractivity molar range must be between 40-130. The last parameter is optional, because in the previous research, the emphasized parameters are the first four. However, the drug scan result shows that firstly, the SAHA standard ligand, and secondly, the modified ligands are in accordance with the five parameters, with the exception of the molecular weight of 2f ligand. We could thus infer that the ligands have successfully passed Lipinski’s Rules.

The oral bioavailability of drugs could be measured by the molecular weight, number of rotatable bonds (n rotb), number of hydrogen bonds (n ON and n OHNH), and the expanse of the drug’s polar surface (TPSA). This set of criteria is called Veber’s rule. The oral bioavailability was marked by small molecular weight (less than 500); also, the number of rotatable bond must be less than 10, the number of hydrogen bond donors and acceptors must be less than 12, and TPSA values less than 140. Table 7 has shown that the SAHA standard ligand and the modified ligands have good oral bioavaibility, with the exception of 2b, 2d, and 2e ligands. Those ligands are still in accordance with Veber’s rule.

The hydrophobicity of drugs could be inferred from Log P value. When its value is increasing, the drug will be more hydrophobic. When the drug is more hydrophobic, then the drug will be able to circulate longer in our body, because it wouldn’t be easy to secrete it. The table 7 shows, that the Log P values of the 1c, 1d, 1f, 2a, 2c, 2d, 2e, and 2f modified ligands are larger than the SAHA standard ligand. It shows that the modified ligands are more hydrophobic than SAHA. Normally, drugs, which interact with enzyme inside human body, have Log P value between 2 and 5 [11]. The table shows that only modified 1b and 1d ligands, which have log P value less than 2.

The drug likeness value of standard and modified ligand shows the fragment content of the drugs. If the drug likeness values are increasing, than it has the same fragment content with existing drugs. From Table 7, it is shown that the drug likeness value of 1a, 1c, 1e, 1f, 2a, 2c, 2d, 2e, and 2f ligands are larger than the SAHA standard ligand. The highest drug likeness values are in 2f ligand. This result tells us, that the modified ligand has the most fragments content of drugs.

The drug score values are the combination of drug likeness, Log P, solubility, molecular weight, and toxicity risk within one useful practical value. It could be used for evaluating the potential of the drug candidate [12]. When the drug score is better, then the compound has a better chance to be a drug candidate. Table 7 shows that only modified 1a and 1e ligands have better drug score than SAHA standard ligand.

### Inhibitor toxicity prediction

This research is using three different softwares to predict Inhibitor toxicity. They are Osiris Property Explorer, Toxtree v2.1.0 and Lazar. All of them have different parameters for determining the toxicity of compounds. The prediction using Osiris Property Explorer was shown in colour codes. The result of toxicity analysis of SAHA standard ligand, first, and second modified ligands is shown in table 8.

**Table 8:** Toxicity of SAHA standard and modified ligand based on Osiris Property Explorer.

Ligand	***Mutagenic***	***Tumorigenic***	***Irritant***	***Reproductive effective***
SAHA	yellow	green	green	green
1a	yellow	green	green	green
1b	red	red	yellow	yellow
1c	yellow	green	green	green
1d	yellow	green	green	green
1e	yellow	green	green	green
1f	yellow	green	green	green
2a	yellow	green	green	green
2b	red	red	yellow	yellow
2c	yellow	green	green	green
2d	yellow	green	green	green
2e	yellow	green	green	green
2f	yellow	green	green	green

Green colour shows the low toxicity tendency, yellow shows the mediocre tendency, and red shows high tendency. Table 8 shows that only 1b and 2b ligands have high toxicity. This happens because they have mutagenic aniline group (C_6_H_5_NH_2_). Other ligands have low or mediocre toxicity.

Inhibitor toxicity could be verified by using Toxtree v2.10, and the result could be seen at table 9.

**Table 9:** Toxicity of SAHA standard and modified ligand based on Toxtree v2.1.0.

Ligand	***Negatif for genotoxic carcinogenity***	***Negatif for nongenotoxic carcinogenity***	***Potential S.Typhiurium TA 100 mutagen based on QSAR***	***Potential carcinogen based on QSAR***
SAHA	yes	yes	no	no
1a	yes	yes	no	no
1b	no	yes	no	no
1c	yes	yes	no	no
1d	yes	no	no	no
1e	yes	yes	no	no
1f	yes	no	no	no
2a	yes	yes	no	no
2b	no	yes	yes	no
2c	yes	yes	no	no
2d	yes	no	no	yes
2e	yes	yes	no	no
2f	yes	no	no	no

Toxtree v2.1.0 determined the toxicity level of compounds based on Benigni and Bossa rules. It stipulated that certain functional groups, which have mutagenic or carcinogenic properties, could influence the toxicity. The table 9 shows that 1b and 2b ligands have tendency to be mutagenic, while 1d, 1f, 2d, and 2f ligands have potential to be carcinogenic. However, when Toxtree software result was compared with QSAR analysis, only 2b ligand has mutagenic potential, and 2d ligand has carcinogenic potential. SAHA standard ligand and 1a, 1c, 1e, 2a, 2c, and 2e modified ligands were in accordance of Benigni and Bossa Rules, because they don’t have potential to be mutagenic or carcinogenic.

Lazar is a software package with functionality of detecting mutagenic or carcinogenic properties based on the functional group similarity with mutagenic or carcinogenic ones. The analysis result of inhibitor toxicity by lazar could be read at table 10.

**Table 10:** Toxicity analysis result by using Lazar.

Ligand	***Mutagenicity***		***Carcinogenicity***		
	
	***Salmonella typhimurium*** **(CPDB)**	***Salmonella typhimurium*** **(Kazius/Bursi)**	***Rodent*** **(*****multiple sex*****)**	***Rat*** **(*****both sex*****)**	***Mouse*** **(*****both sex*****)**
SAHA	no	no	no	no	yes
1a	no	no	no	no	no
1b	no	no	no	no	yes
1c	no	no	no	no	no
1d	no	no	no	no	no
1e	no	no	yes	no	no
1f	no	no	no	no	no
2a	no	no	no	no	yes
2b	no	no	no	no	yes
2c	no	no	no	no	yes
2d	no	no	no	no	yes
2e	no	no	no	no	yes
2f	no	no	no	no	yes

Lazar verified the mutagenicity of compounds by conducting assay test with *Salmonella typhimurium*. Table 10 shows that SAHA standard, first, and second modified ligands have no mutagenic properties. Moreover, the carcinogenicity of compounds was verified by animal testing, with rodent, Rat, and Mouse. Table 10 also shows that SAHA standard ligand has no carcinogenic property toward the three animals. However, the 1e modified ligand has carcinogenic property toward rodent, 1b and other second modified ligands were carcinogenic toward mouse.

### Screening the best ligand based on docking and drug scan

The best ligands for each class II HDACH *Homo sapiens* could be determined based on drug scan and docking analysis [13]. The best ligand for HDAC4, HDAC6, and HDAC7 is 2c. It is because the **ΔG_binding_** and Ki of 2c were the smallest compared with other ligands, with the exception at HDAC 7. The smallest values of **ΔG_binding_** and Ki in HDAC 7 were obtained in 2d and 2f ligand. However, 2d and 2f ligands have bad pharmacological and toxicity attributes, based on drug scan analysis. 2f Ligand is having a molecular weight, which is beyond the threshold of Lipinski’s Rule parameter. Moreover, the halogen group on 2d and 2f ligands could cause carcinogenic property on those ligands. 2c Ligand has good pharmacological attributes based on drug scan analysis, because it is in accordance of Lipinski’s Rule, Veber’s Rule, Log P values, toxicity, drug likeness and drug score threshold.

The best ligand for HDAC 5 is 2a. This ligand has smaller **ΔG_binding_** and Ki compared with 2f, 2e, and 2b ligands. However, based on drug scan analysis, 2a ligand has better pharmacological property. The ligand is in accordance with Lipinski’s rule parameter and oral bioavailability. 2f, 2e, and 2b ligands were not in accordance to those parameters, because of its molecular weight (2f ligand), and the amount of the hydrogen bond (2b and 2e ligands). Moreover, the toxicity of 2f and 2b ligands have mutagenic and carcinogenic tendency, because of the halogen group (2f ligand) and aniline group (2b ligand). The toxicity of 2a ligand is lower than 2f and 2b ligands.

The best ligands for HDAC9 and 10 are 1c. It has the smallest **ΔG_binding_** and Ki compared with the others. 1c ligand has good pharmacological properties, because it is in accordance with Lipinski’s Rule, Veber’s rule, high hydrophobicity, and low toxicity.

The property difference among the best ligands on each class II HDAC *Homo sapiens* was because of the characteristic differences among those enzymes. The difference of catalytic sites of each enzyme causes the interaction tendency of ligands to be different as well.

The screening result shows that every ligand has the same alkyl group, which is cyclohexyl (C_6_H_11_). 2a, 1c, 2c ligands are exceptions. The cyclohexyl group has a high tendency of hydrophobicity, and it could be seen by its log P values. This matter would eventually influence the ability of the group to push the ligand for breaking in the lipid bilayer, in order for the drug to bind stronger with the enzyme. Moreover, the hydrophobic group on ligand would make the interaction between ligand and enzyme run smoothly [4].

### Best ligand 3D visualisation toward class II HDAC *Homo sapiens*

**Figure 6:**
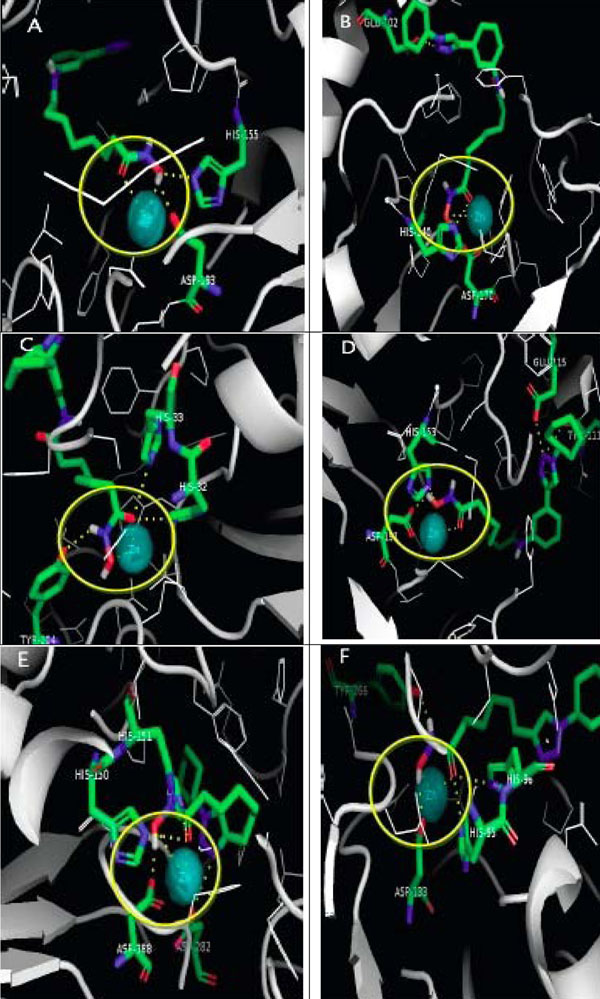
**3D Docking simulation results.** Interaction between: A. 2c ligand and HDAC 4 *Homo sapiens. B.* 2a ligand and HDAC 5 *Homo sapiens. C.* 2c ligand and HDAC 6 *Homo sapiens. D.* 2c ligand and HDAC 7 *Homo sapiens. E.* 1c ligand and HDAC 9 *Homo sapiens. F.* 1c ligand and HDAC 10 *Homo sapiens* are shown. The area with yellow circles shows the best ligand interaction with Zn^2+^ ion as the enzyme catalytic site.

**Figure 7:**
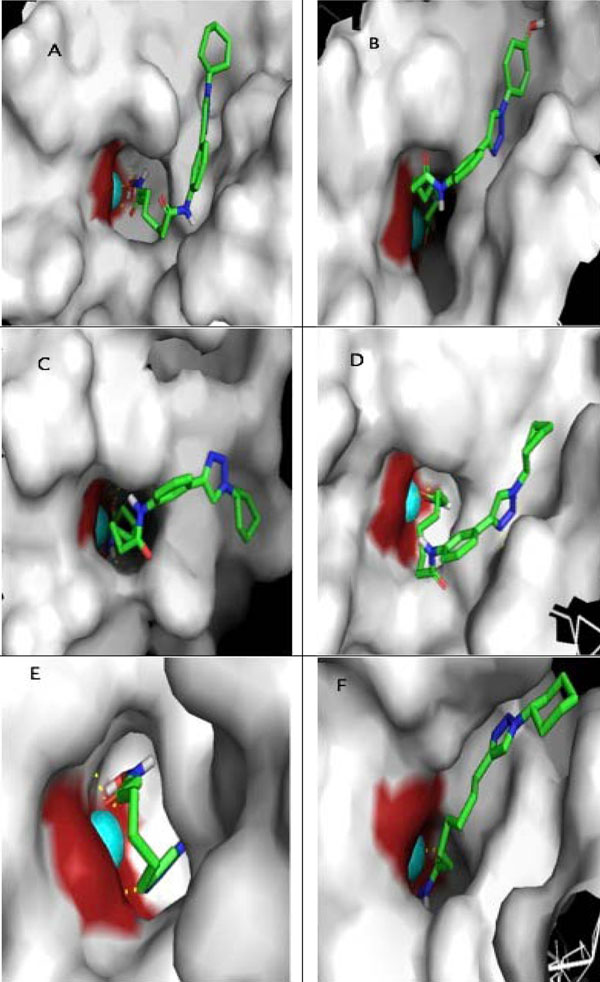
**Surface area of ligand-enzyme interactions.** Interactions are shown between A. 2c ligand and HDAC 4 *Homo sapiens*. B. 2a ligand and HDAC 5 *Homo sapiens*. C. 2c ligand and HDAC 6 *Homo sapiens*. D. 2c ligand and HDAC 7 *Homo sapiens*. E. 1c ligand and HDAC 9 *Homo sapiens*. *F. 1c ligand and HDAC 10 Homo sapiens.* These show that the cavity of enzyme is deep enough for ligand binding. The depth makes it necessary to utilize certain ligands with long aliphatic ring, in order for it to enter the cavity, and interact with Zn^2+^ ion. Hydrophobic cap group is necessary as well, in order to guarantee the interaction between ligand’s cap groups with enzyme’s surface area.

Visualisation is the final step of SAR study. The screening result of the best ligand conformation visualization was conducted by using PyMOL [14]. The best ligand interaction toward class II HDAC *Homo sapiens* in 3D could be seen at figures 6. The area with yellow circles shows the best ligand interaction with Zn^2+^ ion as the enzyme catalytic site. The figure 7 shows the surface area of ligand-enzyme interaction, where it shows that the cavity of enzyme is deep enough. The depth makes it necessary to utilize certain ligand with long aliphatic ring, in order for it to enter the cavity, and interact with Zn^2+^ ion [15]. Hydrophobic cap group is necessary as well, in order to guarantee the interaction between ligand’s cap groups with enzyme’s surface area. Our future step to improve our validation method is by using Lead-Finder benchmarking tools. [16]

## Conclusions

The docking result of SAHA standard, first, and second modified ligands toward class II HDAC *Homo sapiens* shows that those ligands have same type of interaction toward class II HDAC *Homo sapiens*, which are utilizing O atom on C=O group, and its –OH group is binding to the Zn^2+^ ion. The ion is the enzyme’s catalytic site. Beside, the SAHA standard and modified ligands are bonded via hydrogen bonds with amino acid residues around the enzymatic catalytic sites. Then, the analysis of **ΔG_binding_** and Ki show that every first and second modified ligands have smaller **ΔG_binding_** and Ki than SAHA standard ligand. It could be inferred that every first and second modified ligand has better binding affinity than SAHA standard ligand. The complex of first and second modified ligand with class II HDAC *Homo sapiens* are much more preferable compared with the SAHA standard ligand.

Every modified ligand has good pharmacological properties, and it could be inferred by its accordance with Lipinskis Rule, Veber’s Rule, hydrophobicity based on log P value, and good drug likeness and drug score. However, 2b, 2e, and 2f ligands are exceptions. Moreover, only 1a, 1c, 1e, 2a, 2c, and 2e ligands have low toxicity value.

The best ligands according to the binding energy and drug scan analysis are 1c and 2c ligands, which have the same alkyl groups, the cyclohexyl (C_6_H_11_). In this end, our SAR study has proven that 1c and 2c inhibitors are the best inhibitor as alternatives of SAHA.

## Competing interests

The authors declare that they have no competing interests.

## Authors' contributions

USFT supervised the research, BN was working on the technical and experimental details, AAP prepared the English manuscript; re-verified the data; and giving critical suggestion for the whole experiments.
